# On the Use and Abuse of Alcoholic Liquors, in Health and Disease

**Published:** 1850-07

**Authors:** 


ART. IX.—*On the Use and Abuse of Alcoholic Liquors, in Health and Disease. Prize Essay.* By WILLIAM B. CARPENTER, M. D., F. R. S., F. G. S., author of "Principles of Human Physiology," etc., etc. Philadelphia: Lea & Blanchard. 1850.

This is a prize essay, chosen from among fifteen MS. essays, on the Use and Abuse of Alcoholic Liquors. It was written in answer to certain queries on the subject, and one hundred guineas were offered for the best essay. The committee of adjudication were Drs. Forbes, Roupell & Grey.

We have no great predilection for prize essays on any subject, for they rarely meet expectation. The Bridgewater Treatises are a notable example of this fact, and we think Dr. Carpenter's treatise is not an exception to the rule. In reading it, we have been astonished that the author of such a work as the "Principles of Human Physiology," could have contrived to put together such an *omnium gatherum* as this. It possesses but little of that force, directness, and interest that we should have looked for from an author of Dr. Carpenter's reputation.

There is an immense waste of benevolence in this world, under, what should be called, misdirected philanthropy, and men seem to learn no wisdom from experience. From the clear and simple truths of christianity, men turn to their own schemes of reform, and expect great results from the goodness of their intentions. The vice of drunkenness prevails in the land, and men crowd upon it as though it was the only one to be destroyed. Societies are formed for its suppression, money is raised, tracts printed and distributed until they cannot find readers; prizes are offered for new excitants and they spring forth for the lucre; lecturers perambulate the country; reformed drunkards are seized and placed before the people as teachers; a round of excitement is taken, and when the storm lulls, the reformed inebriate feels his old hankerings returning upon him, and in the stillness of solitude, he flies to what he had just been denouncing in the crowded assembly. If we calmly compare the results with the outlay of effort, we find the outlay greatly exceeds the results. And thus it will ever be as long as men consider their wisdom superior to Heaven's ordinances. The church was established as the means of reforming the world, and having failed in her duties, she now asks the earth to help her, and her members run, and join those who do not know christianity, in efforts to reform the world from its various and multiform iniquities. These things should not be so.

We agree with Dr. Carpenter, however, in his belief that physicians should have nothing to do with intoxicating drinks. They should not prescribe anything of the kind, for any purpose whatever. There is no physician, who is in any degree intimate with the resources of the materia medica, that can have any valid excuse for directing alcoholic drinks for his patients. The resources of medicine are ample without anything of the kind.

We have referred to the character of this work, and of the disappointment we have experienced in reading it. The author has made his work a species of patch work, and uses the experimental investigations of others with great freedom, without attempting any of his own, or the verification of those he uses. The work is, therefore, a series of paths well beaten by others. As an adjunct to the temperance reform, we do not think it comparable to Macknish's "Anatomy of Drunkenness." That an author of the powers of Dr. Carpenter, should have permitted such a work as this to go forth, under the use of his name is surprising. Neophytes in temperance literature may derive some benefit from a perusal of the work, but those who have studied works devoted to the cause of temperance, will rise from this but little, if any improved.

